# Cortical and Subcortical Network Dysfunction in a Female Patient With *NEXMIF* Encephalopathy

**DOI:** 10.3389/fneur.2021.722664

**Published:** 2021-09-09

**Authors:** Maria Cristina Cioclu, Antonietta Coppola, Manuela Tondelli, Anna Elisabetta Vaudano, Giada Giovannini, S. Krithika, Michele Iacomino, Federico Zara, Sanjay M. Sisodiya, Stefano Meletti

**Affiliations:** ^1^Department of Biomedical, Metabolic, and Neural Science, University of Modena and Reggio Emilia, Modena, Italy; ^2^Department of Neuroscience, Reproductive and Odontostomatological Sciences, Federico II University, Naples, Italy; ^3^Neurology Unit, OCB Hospital, Azienda Ospedaliera Universitaria di Modena, Modena, Italy; ^4^PhD Program in Clinical and Experimental Medicine, University of Modena and Reggio Emilia, Modena, Italy; ^5^Department of Clinical and Experimental Epilepsy, UCL Queen Square Institute of Neurology, London, United Kingdom; ^6^The Chalfont Centre for Epilepsy, Chalfont-St-Peter, Bucks, United Kingdom; ^7^School of Life Sciences, Anglia Ruskin University, Cambridge, United Kingdom; ^8^Unit of Medical Genetics, IRCCS Giannina Gaslini Institute, Genova, Italy; ^9^Department of Neurosciences, Rehabilitation, Ophthalmology, Genetics, Maternal and Child Health, Faculty of Medical and Pharmaceutical Sciences, University of Genoa, Genova, Italy

**Keywords:** *NEXMIF*, non convulsive status epilepticus, developmental and epileptic encephalopathy, epilepsy, eyelid myoclonia with absences, fMRI

## Abstract

The developmental and epileptic encephalopathies (DEE) are the most severe group of epilepsies. Recently, *NEXMIF* mutations have been shown to cause a DEE in females, characterized by myoclonic–atonic epilepsy and recurrent nonconvulsive status. Here we used advanced neuroimaging techniques in a patient with a novel *NEXMIF de novo* mutation presenting with recurrent absence status with eyelid myoclonia, to reveal brain structural and functional changes that can bring the clinical phenotype to alteration within specific brain networks. Indeed, the alterations found in the patient involved the visual pericalcarine cortex and the middle frontal gyrus, regions that have been demonstrated to be a core feature in epilepsy phenotypes with visual sensitivity and eyelid myoclonia with absences.

## Introduction

The developmental and epileptic encephalopathies (DEEs) are the most severe group of epilepsies in which frequent epileptic activity, in addition to the underlying etiology, contributes to developmental impairment, with onset typically in infancy or childhood (1). At least 50% of the DEEs have a genetic cause (2), and there is significant etiological overlap with other neurodevelopmental disorders such as intellectual disability (ID) and autism spectrum disorder (3).

The Neurite EXtension and MIgration Factor gene (*NEXMIF*), previously called *KIAA2022* (OMIM 300524), is an X-linked gene thought to play an important role in early brain development (4–7). Pathogenic *NEXMIF* variants were first identified in males with non-syndromic X-linked ID with poor or absent speech, subtle dysmorphic features, and sometimes epilepsy (8, 9). Subsequently, affected females have been described (10–14) and recently a large multicentric study outlined the epilepsy phenotype of affected females (15) which is consistent with a generalized DEE characterized by myoclonic–atonic epilepsy overlapping with eyelid myoclonia with absence. Notably a considerable proportion of affected females present prolonged seizures characterized by absence status with eyelid myoclonia (15, 16). We report a female patient with a *de novo NEXMIF* pathogenic variant and recurrent prolonged episodes of absence status with eyelid myoclonia. In order to evaluate the consequences of *NEXMIF* mutation at brain structural and functional MRI level (17) two different studies were carried out comparing the patient with respect to patients with genetic generalized epilepsy (GGE; formerly idiopathic) and healthy controls (HC).

## Patient and Methods

The patient is a 28-year-old woman, with a drug-resistant epilepsy starting at the age of 9 years with recurrent episodes of prolonged non-convulsive status epilepticus (NCSE) characterized by mydriasis, eyelid myoclonia and reduced responsiveness to environmental stimuli with a frequency of 1–2 episodes/month. She was born pre-term (8th gestational month) from a dichorionic diamniotic twin pregnancy, from unrelated parents. Family history is unremarkable for epilepsy, febrile seizures, and any other neurological condition. Her developmental milestones were slightly delayed (she started walking at 16 months and talking at 24 months). She afterwards achieved borderline intellectual functioning (full-scale IQ 75 at the age of 24 years; WAIS) with difficulties in visuo-spatial information processing and dyspraxia. The patient also presented night terrors and enuresis until late adolescence. Her neurological examination is unremarkable.

During NCSE, which may last up to 48 h, the patient first complains of an epigastric discomfort, followed by headache and subsequent clouding of consciousness when she becomes progressively more unaware and detached from the environment. During this phase, the patient has mydriasis and presents subcontinuous eye-blinking. EEGs recorded during NCSE show a continuous, diffuse, spike- and poly-spike and wave-discharge, worsened by eye closure, hyperventilation and by intermittent photic stimulation (Supplementary Figure 1). Characteristically eye closure induces the paroxysmal discharges while these tend to reduce or disappear when the patient is asked to open her eyes/stare or to perform a mental task. Beyond these episodes the patient's EEG background activity appears normal, with only occasional diffuse sharp waves, mainly elicited by eye closure.

Various anti-seizure medications (ASM) in numerous combinations were tried without achieving substantial benefit. She was first started on valproic acid, with clonazepam, and then switched to topiramate because of lack of efficacy. Lamotrigine, lacosamide, zonisamide, acetazolamide and ethosuximide were ineffective or even detrimental. A vagal nerve stimulator was implanted at the age of 25 years. At the time of this report, the patient is on valproate (1,000 mg), lamotrigine (150 mg) and brivaracetam (150 mg) but continues to have recurrent NCSE even if more rarely than in the past.

Extensive metabolic and endocrinological screening showed no significant abnormalities except for secondary amenorrhea. Liver and renal functions are normal, and no alterations were found on abdominal ultrasound and echocardiography. Brain structural MRI shows no abnormality.

### Genetic Testing

A NGS exome sequencing was performed on genomic DNA from the patient and her parents by using the Nextera Rapid Capture Exomes kit and massively parallel sequencing (Illumina, PE 2 × 150). Sequence mapping and variant calling were performed using GATK software. Variants with certain or probable pathogenic significance based on ACMG guidelines were validated by Sanger sequencing.

The analysis revealed the presence of the chrX-73962221-C- (GRCh37/hg19 assembly) variant in the *NEXMIF* gene (c.2171delG, NM_001008537, p.S724MfsTer5) in the heterozygous state. This variant is not reported in the international registry of mutations published in the literature (ClinVar) and is not present in the human polymorphism databases (GnomAD, dbSNP147). The segregation analysis showed the variant was *de novo*. The variant is predicted to have pathogenic consequences according to ACMG criteria. Additionally, at the protein level, the p.S574MfsTer5 variant affects an evolutionarily highly conserved amino acid according to *in-silico* tool GERP++ score 5.73. The variant is predicted to cause a non-sense mediated decay and a premature truncation with effect on protein function according to *in-silico* tools (Sift score 0.858, Provean score −5.16 and Mutation taster).

### Structural MRI Study

For comparison, 20 matched females with GGE and 20 matched healthy females (HC) were recruited. The mean age of the GGE group was 22.74 years old; that of the HC group was 28.45 (*p* > 0.05). All GGE patients had normal structural brain MRI on conventional diagnostic protocol at 3 Tesla and no intellectual disability (full-scale *IQ* > 80) or psychiatric comorbidities. Since the patient was under treatment with valproate at the time of the study, and valproate has been demonstrated to be associated with subcortical atrophy and posterior cortical thinning (18, 19), we included in the GGE control group only females (selected from our MRI research database) that were already on treatment with valproate at the time of the MR imaging study. The HC had no history of neurological diseases or past valproate use, or family history of epilepsy, and had normal structural neuroimaging. Moreover, all controls had a normal EEG, since they were recruited for previous EEG-fMRI co-registration study protocols by our group. The details of MRI acquisition and post-processing analyses are reported in Supplementary Material.

#### MRI Cortical Thickness and Subcortical Volume Analyses

Scans were analyzed using a standardized image toolbox (Freesurfer, version 5.0) (20), quality assurance (outlier detection based on inter quartile of 1.5 standard deviations along with visual inspection of segmentations), and statistical methods. Visual inspections of subcortical and cortical segmentations were conducted following standardized ENIGMA protocols (http://enigma.usc.edu), used in prior genetic studies of brain structure (21, 22), large-scale case-control studies of epilepsy (23) and neuropsychiatric illnesses (24, 25).

Statistical analyses were performed using SPSS software 26.0 (IBM, Chicago, IL). To compare cortical measures between the proband and each group, we conducted the Crawford's modified independent sample *t*-test using the program singlims.exe (https://homepages.abdn.ac.uk/j.crawford/pages/dept): this tests whether a patient's score is significantly below controls, thus providing a point estimate of the abnormality of the patient's score (i.e., it estimates the percentage of the control population exhibiting a lower score), accompanying confidence limits on this quantity, and results with point and interval estimates of effect sizes (26). Percentile calculation for each variable was performed to inspect case's distribution values in comparison to HC and GGE.

### Functional MRI Study

Patients (the proband and GGE) and controls were investigated by means of a task-related EEG-fMRI protocol in order to elucidate brain activity related to eye-closure condition. In this second study the GGE population consisted of 14 patients (13 females, mean age = 24.9 years, mean age of epilepsy onset= 12.6 years). The healthy control group consisted of 16 subjects (12 females, mean age = 28 years). The experimental protocol and EEG-fMRI data pre-processing and analysis have been extensively described previously by our group (see Supplementary Materials) (27).

## Results

### Cortical Thickness and Subcortical Volumes

Subcortical structural comparison between the proband and HC group showed volume reduction in the right thalamus (*p* = 0.02), right amygdala (*p* = 0.04), and left caudate (*p* = 0.04). Cortical thickness analyses showed that the patient had reduced cortical thickness in several brain regions in comparison to HC, including left (*p* = 0.005) and right caudal middle frontal gyrus (*p* < 0.001), left fusiform (*p* = 0.03) and left inferior parietal gyrus (*p* = 0.004), left (*p* = 0.01), and right (*p* = 0.03) lateral occipital gyrus, and left lingual gyrus (*p* = 0.03; Table 1).

**Table 1:** Significant differences in subcortical volumes and cortical thickness in the patient compared to GGE sample.

	**GGE**			**Significance test**		**Estimated percentage of the GGE population obtaining a lower score than the case**			**Estimated effect size**		
	**Mean**	**Standard deviation**	**Proband**	***t***	***p***	**Point**	**95% CI**		**Point**	**95% CI**	
**Subcortical structures volumes (mm** ^**3**^ **)**									
R thalamus	6479.69	704.78	5236.7	−1.721	**0.05**	5.07	0.68	14.77	−1.764	−2.463	−1.046
**Cortical thickness (mm)**									
L caudal middle frontal	2.50	0.22	1.938	−2.528	**0.02**	1.02	0.02	4.88	−2.564	−3.51	−1.656
L lingual	2.02	0.13	1.77	−1.952	**0.05**	3.29	0.28	11.06	−2	−2.759	−1.223
R caudal middle frontal	2.47	0.24	1.847	−2.673	**0.01**	0.75	0.01	3.89	−2.739	−3.7	−1.763
R pars triangularis	2.58	0.26	1.992	−2.215	**0.03**	1.96	0.09	7.75	−2.269	−3.1	−1.421
R precentral	2.31	0.15	1.926	−2.537	**0.02**	1	0.02	4.82	−2.6	−3.522	−1.662
R superior temporal	2.89	0.20	2.431	−2.196	**0.04**	2.03	0.1	7.96	−2.25	−3.076	−1.407

*p-values are written in bold*.

Subcortical structural comparison between proband and GGE group showed that the case had volume reduction in the right thalamus (*p* = 0.05). Cortical thickness analyses showed that the patient had reduced cortical thickness in several brain regions in comparison to GGE, including left (*p* = 0.02) and right caudal middle frontal gyrus (*p* = 0.01), and the left lingual gyrus (*p* = 0.05; Table 2).

**Table 2:** Significant difference in subcortical volumes and cortical thickness in proband compared to healthy controls.

	**Healthy controls sample**			**Significance test**		**Estimated percentage of the HC population obtaining a lower score than the case**			**Estimated effect size**		
	**Mean**	**Standard deviation**	**Proband**	***t***	***p***	**Point**	**95% CI**		**Point**	**95% CI**	
**Subcortical structures volumes (mm** ^**3**^ **)**									
R thalamus	6785.84	624.26	5,237	−2.423	**0.02**	1.27	0.03	5.74	-2.482	−3.372	−1.577
L caudal	3624.45	448.02	2,614	−2.2	**0.04**	2.01	0.1	7.91	-2.254	−3.081	−1.411
R amygdala	2028.33	216.60	1,545	−2.187	**0.04**	2.07	0.1	8.06	-2.241	−3.064	−1.401
**Cortical thickness (mm)**									
L caudal middle frontal	2.50	0.17	1.938	−3.11	**0.005**	0.28	0	1.85	-3.187	−4.277	−2.084
L fusiform	2.71	0.13	2.398	−2.327	**0.03**	1.55	0.05	6.6	-2.385	−3.247	−1.506
L inferior parietal	2.36	0.13	1.923	−3.228	**0.004**	0.22	0	1.5	-3.308	−4.432	−2.169
L lateral occipital	2.22	0.15	1.849	−2.649	**0.01**	0.79	0.01	4.05	-2.714	−3.668	−1.745
L lingual	2.02	0.11	1.77	−2.342	**0.03**	1.51	0.05	6.46	-2.4	−3.266	−1.517
L middle temporal	2.88	0.17	2.397	−2.813	**0.01**	0.55	0	3.1	-2.882	−3.884	−1.866
L pars opercularis	2.47	0.17	2.104	−2.067	**0.05**	2.63	0.18	9.5	-2.118	−2.908	−1.31
L postcentral	2.01	0.13	1.697	−2.402	**0.02**	1.33	0.04	5.91	-2.462	−3.345	−1.562
L superior temporal	2.90	0.20	2.391	−2.62	**0.01**	0.84	0.01	4.24	-2.684	−3.63	−1.723
L supramarginal	2.46	0.16	2.116	−2.135	**0.04**	2.3	0.13	8.66	-2.188	−2.996	−1.361
R caudal middle frontal	2.52	0.18	1.847	−3.904	** <0.001**	0.04	0	0.39	-4	−5.329	−2.659
R lateral occipital	2.18	0.11	1.92	−2.307	**0.03**	1.62	0.06	6.8	-2.36	−3.22	−1.491
R pars triangularis	2.49	0.19	1.992	−2.711	**0.01**	0.69	0.008	3.66	-2.778	−3.75	−1.792
R peri calcarine	1.64	0.12	1.356	−2.484	**0.02**	1.12	0.02	5.23	-2.545	−3.452	−1.623
R precentral	2.39	0.15	1.926	−3.058	**0.006**	0.32	0.001	2.041	-3.133	−4.207	−2.045
R superior temporal	2.99	0.16	2.431	−3.416	**0.002**	0.14	0	1.05	-3.5	−4.681	−2.306

*p-values are written in bold*.

**Figure 1:**
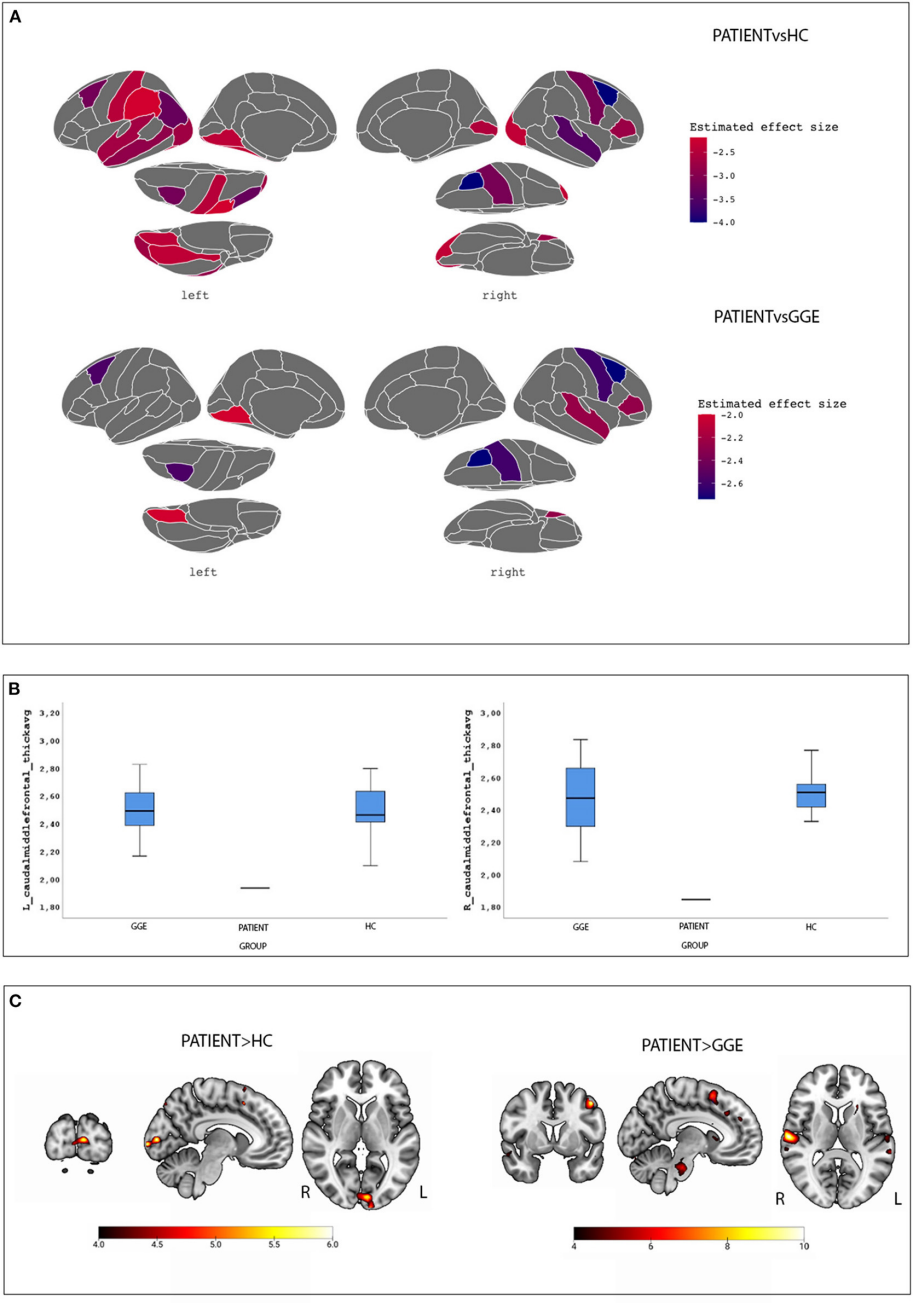
Morphometric and Functional results. **(A)** surface brain templates depicting regions of cortical thinning in the proband compared to HC (top images) and GGE (bottom images). Brain statistics were displayed using the ggseg and ggseg3d packages integrated into the software R environment using the Desikan-Killiany cortical atlas (28). **(B)** Percentile distribution of cortical thickness for the left and caudal middle frontal gyri in the case compared to controls and GGE; see text for details. **(C)** BOLD maps related to the eye closure conditions in the patient compared to controls (left images) and GGE (right images). Results are overlaid into the MNI152 template as provided by the MRIcroGL toolbox.

Figure 1A shows surface brain template depicting regions of cortical thinning in the proband compared to HC and GGE. Percentile distribution confirmed that in the patient, right and left caudal middle frontal gyri cortical thickness had values below IQR and extreme values in comparison to both HC and GGE (Figure 1B). Supplementary Table 1 show percentile distribution for the patient in comparison to all HC and GGE.

### Brain Correlates of Eye-Closure

A total of 13 voluntary eyes closure conditions were recorded. Compared to HC, the patient demonstrated increased BOLD signal changes at the left cuneus. When compared to GGE, a diffuse network appeared encompassing the left precentral gyrus, the left basal ganglia, the bilateral superior temporal gyrus, the right inferior frontal gyrus and the pons (Figure 1C; Supplementary Table 2). No significant BOLD changes were observed in the opposite comparisons (i.e., HC and GGE vs. the proband).

## Discussion

*NEXMIF* plays an important role in neural circuit formation during development (4–8). Knockdown of *NEXMIF* leads to dramatic impairment in neurite outgrowth, with a particular impact on the lengths of dendrites and axons (9). To our knowledge, this is the first study to attempt to identify whether pathogenic *NEXMIF* variants induce alterations in brain structure or in functional networks in humans. In summary, compared to other populations, the studied patient shows a thinning of the prefrontal cortex and in particular of the middle frontal gyrus, of the temporal lobe cortex (including the fusiform gyrus) and of pericalcarine visual cortex. Consistently, these areas have shown functional alterations (increase of BOLD signal compared to controls) in the condition of eye closure: this finding is of interest because this pattern of functional activation was previously documented in patients with a clinical phenotype characterized by eyelid myoclonia and absences (27). In fact, the patient, although not having the clinical phenotype typical of Jeavons syndrome, demonstrated prolonged NCSE episodes characterized by absences with eyelid myoclonia.

Here the use of advanced neuroimaging techniques in a specific genetic phenotype revealed brain structural and functional changes that can bring the clinical phenotype to alteration within specific brain networks, and especially in networks physiologically involved in several visuomotor function, including the motor control of eye-closure and eye-movements, and attention to visual targets. Notably, it is not possible to determine whether the observed morphometric alterations are ascribable to the dysfunction of the NEXMIF gene primarily, or what role has the repetition of prolonged NCSE on these regions. Indeed, this study needs replication in both males and females carrying pathogenic gene mutations in NEXMIF gene to come to the conclusion that the observed network alterations are gene specific, or mutation mediated effects, or a feature of the association of epileptic seizure phenotype of eyelid myoclonia with absences. To note, it is unlikely that the observed structural/functional MRI changes are result of single gene defect but may be the consequence of effects mediated by more than one gene involved in the development of visuomotor networks. Of course, the results obtained should be considered with caution and reflect patient-specific brain changes. That said, the study has identified consistent alterations in cortical/subcortical morphometry and functional imaging thus providing a link between the genetic alteration and *in vivo* brain functioning/morphology.

## Data Availability Statement

The datasets presented in this study can be found in online repositories. The names of the repository/repositories and accession number(s) can be found below: Dryad [https://doi.org/10.5061/dryad.kwh70rz49].

## Ethics Statement

The studies involving human participants were reviewed and approved by Comitato Etico Provinciale, Azienda Ospedaliero-Universitaria di Modena (study no. 80/10 and 268/15). Written informed consent to participate in this study was provided by the participants' legal guardian/next of kin. Written informed consent was obtained from the individual(s) for the publication of any potentially identifiable images or data included in this article.

## Author Contributions

MC, AC, AV, GG, and SM contributed to the conception of the subject of the manuscript. MC, AC, and SM searched the patient files, interpreted literature, and wrote the manuscript. AC, MI, FZ, SK, and SS contributed to the genetic analysis and genetic interpretation of data. MT, AV, and SM performed the fMRI data analysis. AC, GG, SS, and SM revised the manuscript.

## Funding

This research was partly funded by a Grant on Genetic Epilepsies issued by Fondazione LICE to AC. SS is supported by the Epilepsy Society.

## Conflict of Interest

AC has received research grant support from the Ministry of Health (MOH) and has received personal compensation as scientific advisory board member for EISAI, BIAL, and GW pharmaceutical Company. SM received research grant support from the Ministry of Health (MOH); has received personal compensation as scientific advisory board member for UCB and EISAI. The remaining authors declare that the research was conducted in the absence of any commercial or financial relationships that could be construed as a potential conflict of interest.

## Publisher's Note

All claims expressed in this article are solely those of the authors and do not necessarily represent those of their affiliated organizations, or those of the publisher, the editors and the reviewers. Any product that may be evaluated in this article, or claim that may be made by its manufacturer, is not guaranteed or endorsed by the publisher.
