# Supplementary material for: Cortical and Subcortical Network Dysfunction in a Female Patient With NEXMIF Encephalopathy
Source: Front Neurol. 2021 Sep 9;12:722664. doi: 10.3389/fneur.2021.722664 (PMC8459922; doi:10.3389/fneur.2021.722664)
Supplement: Supplementary file 1 [file Table_1.docx]

Supplementary Material

# Cortical and subcortical networks dysfunction in a female patient with NEXMIF encephalopathy

# Maria Cristina Cioclu^1^, Antonietta Coppola^2^, Manuela Tondelli^3^, Anna Elisabetta Vaudano^3^, Giada Giovannini^1,3,4^, S Krithika^5,6,7^, M. Iacomino^8^, Federico Zara^8,9^*,* Sanjay M. Sisodiya^5,6^, Stefano Meletti^1,3^

# Supplementary Methods

# *MRI acquisition.* Three-dimensional (3D) T1-weighted MRI images were acquired using a 3 Tesla Philips Intera MRI scanner (Best, The Netherlands). A SPGR pulse sequence [echo time (TE) = 4.6, repetition time (TR) = 9.9ms] was used. One hundred seventy contiguous sagittal slices were acquired (voxel size = 1 × 1 × 1 mm) and field of view was 240mm with a matrix size of 256 × 256 × 170. A T2-weighted axial scan was also acquired to allow visual determination of vascular burden or tissue abnormalities.

# *MRI cortical thickness and subcortical volume analyses.* The pipeline involves removal of non-brain tissue, automated Talaraich transformation, segmentation of white matter and grey matter, tessellation of grey/white matter boundary, automated correction of topology defects, surface deformation to form the grey/white matter boundary and grey/cerebrospinal fluid boundary, and parcellation of cerebral cortex. Cortical thickness estimates were calculated as the distance between the grey/white matter border and the pial surface at each vertex. Single value labels extracted based on an automatic algorithm (1, 2) were calculated (34 regions for each hemisphere). Subcortical volumes were calculated with FreeSurfer's automated procedure for volumetric measures. Each voxel in the normalized brain volume was assigned to one label using a probabilistic atlas obtained from a manually labelled training set (3). The labels adopted for the analysis included the putamen, caudate nucleus, globus pallidus, nucleus accumbens, thalamus, amygdala, hippocampus, and the ventricular system.

# *EEG-fMRI protocol*. Scalp EEG was recorded by means of a 32-channel MRI-compatible EEG recording system simultaneously with fMRI acquisitions (3T Philips scan; 30 axial slices; repetition time [TR]/echo time [TE]53.000/50 milliseconds). Patients and controls were constantly observed and recorded by means of a small camcorder positioned on the head coil inside the scanner pointing to the subject’s face to obtain a split-screen video-EEG documentation acquired continuously during the fMRI recording. Three fMRI runs per subjects were acquired (60 volumes each) during which a block-design task of 30 seconds of eyes-open (EO) and eyes-closed (EC) conditions was performed (in total 16 eyes-closed and eyes-open conditions). At first level analysis, one model was built for each subject including as regressor the EC and EO conditions, the blinking (all three treated as stick functions), the physiological activity (modelled as variable duration blocks), and the motion (interscan realignment parameters and their Volterra expansion) as confounds. All the regressors were convolved with the canonical hemodynamic response function, and its temporal and dispersion derivatives. A single t contrast (p<0.05, corrected for multiple comparisons; family-wise error [FWE]) image was generated per subject from the first-level analysis in relation to each regressor of interest. Using the statistical images resulting from single-subject contrasts, two second level 2-sample t-tests were carried out to compare the patient versus healthy controls and versus GGE respectively for the EC condition. A double statistical threshold (voxel-wise p < 0.001 and spatial extent) was adopted to achieve a combined significance, corrected for multiple comparisons, of α < 0.05, as computed by 3dClustSim AFNI routine, using the “-acf” option https://afni.nimh.nih.gov/pub/dist/doc/program_help/3dClustSim.html).

## Supplementary Figures


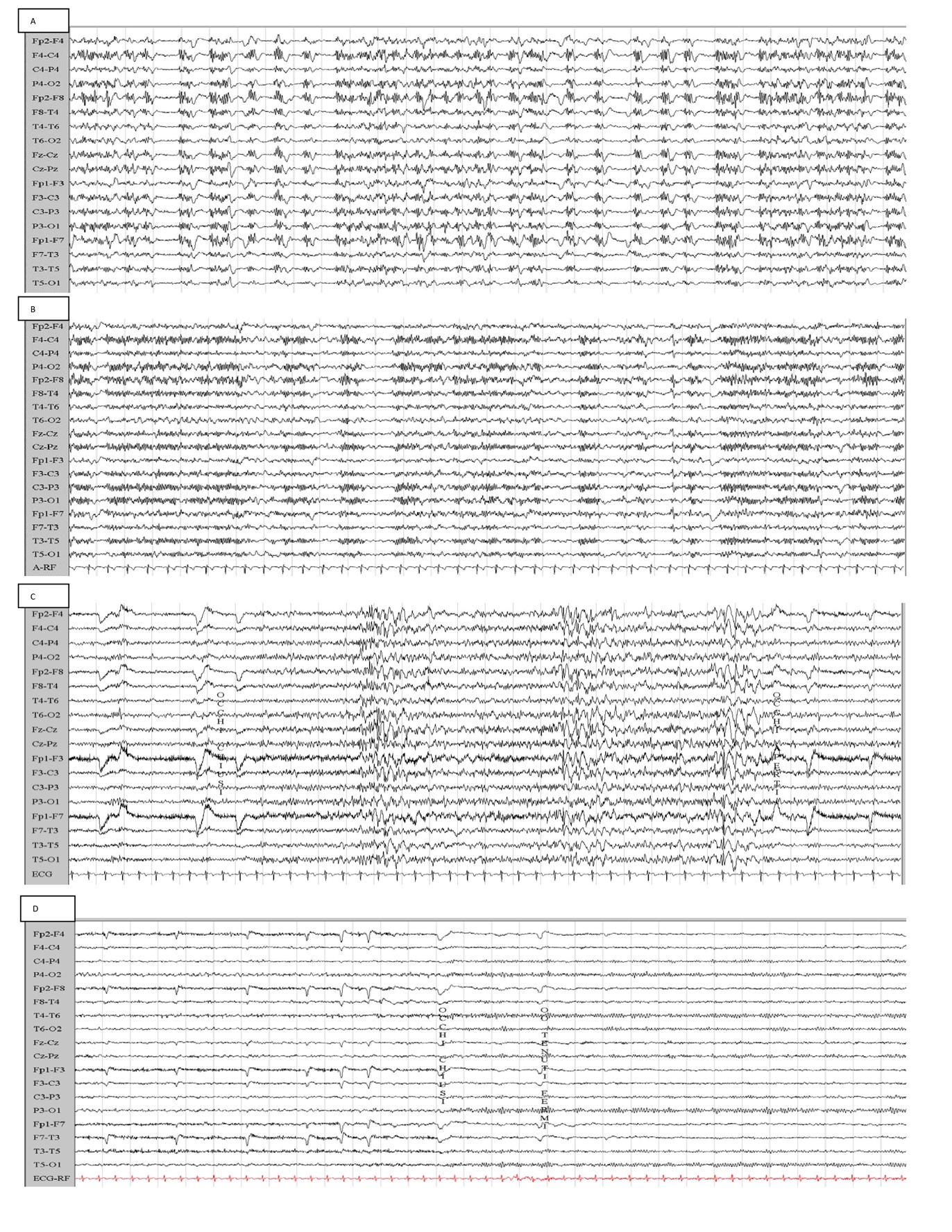
**Supplementary figure 1. Ictal and interictal features of the EEG.** A. shows a typical patient’s absence status characterized by continuous generalized fast polispikes interrupted by brief period of low voltage EEG. B. Status evolution in prolonged runs of fast activities over the central region after benzodiazepine i.v. administration. C. shows the appearance of fast polispikes and generalized spikes and waves after closing the eyes that are interrupted by opening the eyes. D. normal interictal EEG.

# Supplementary Tables


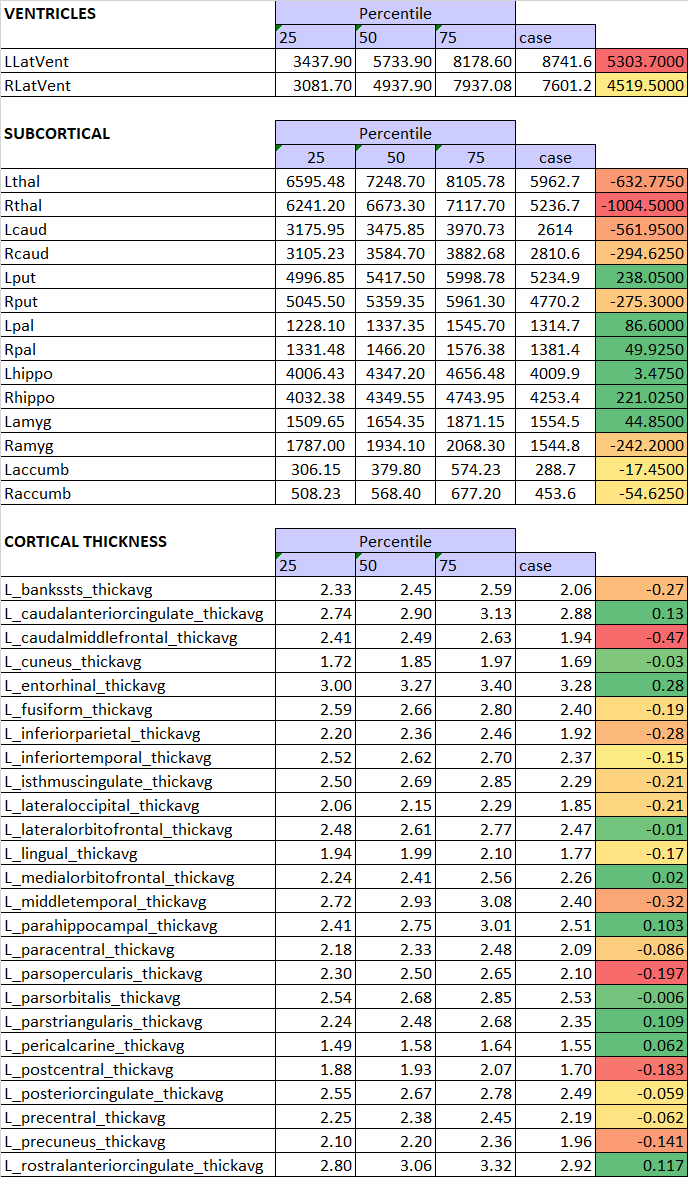


**Supplementary Table 1.** Percentile distribution for the case in comparison to all HC and IGE considered together. Red colour scale is used to highlight values distribution: from lowest, red, to closer, yellow, to the 25° percentile values; in green, values over or equal to 25° percentile.

| ***NEXMIF > HC*** | | | | | | | |
| --- | --- | --- | --- | --- | --- | --- | --- |
|  | **Brain region**  **Cluster maxima** | **Talairach coordinates (mm)** | | | | **Z-scores** | |
|  |  | **x** | **y** | **z** | |  |  |
| **Eye Closure** | Left Cuneus | -12 | -102 | 0 | | 4.58 | |
| ***NEXMIF > GGE*** | | | | | | | |
|  | **Brain region**  **Cluster maxima** | **Talairach coordinates (mm)** | | | | | **Z-scores** |
|  |  | **x** | **y** | | **z** | |  |
| **Eye Closure** | Left Precentral gyrus (BA6)  Right Superior Temporal Gyrus (BA42)  Pons  Right Inferior Frontal Gyrus (BA47)  Left Superior Frontal Gyrus (BA6)  Left Superior Temporal Gyrus (BA21) | -48  64  -4  34  -10  -60 | 0  -14  -24  26  32  -14 | | 50  12  -34  -4  54  -4 | | 5.36  5.03  4.55  4.54  4.23  4.10 |

**Supplementary Table 2. Significant BOLD differences resulted from comparisons between NEXMIF patient and other groups**. Results of the comparison between NEXMIF and healthy controls (HC) (3dClustSim corrected: cluster size threshold k > 166, corrected at α < 0.05) and genetic generalized epilepsy (GGE) (3dClustSim corrected: cluster size threshold k > 239, corrected at α < 0.05). Main cluster grey matter coordinates are given as Talairach coordinates and automatically labeled using Talairach Daemon V1.1 (Research Imaging Center, University of Texas Health Science Center at San Antonio). Z-scores are reported for local voxel maxima.

References

Desikan RS, Ségonne F, Fischl B, Quinn BT, Dickerson BC, Blacker D, et al. An automated labeling system for subdividing the human cerebral cortex on MRI scans into gyral based regions of interest. *Neuroimage.* (2006) 31(3):968-80. doi: 10.1016/j.neuroimage.2006.01.021.

Fischl B, van der Kouwe A, Destrieux C, Halgren E, Ségonne F, Salat DH, et al. Automatically parcellating the human cerebral cortex. *Cereb Cortex*. (2004) 14(1):11-22. doi: 10.1093/cercor/bhg087.

Fischl B, Salat DH, Busa E, Albert M, Dieterich M, Haselgrove C, et al. Whole brain segmentation: automated labeling of neuroanatomical structures in the human brain. *Neuron*. (2002) 33(3):341-55. doi: 10.1016/s0896-6273(02)00569-x.
